# Supplementary material for: The Composition and Antioxidant Activity of Bound Phenolics in Three Legumes, and Their Metabolism and Bioaccessibility of Gastrointestinal Tract
Source: Foods. 2020 Dec 7;9(12):1816. doi: 10.3390/foods9121816 (PMC7762431; doi:10.3390/foods9121816)
Supplement: Supplementary file 1 [file foods-09-01816-s001.pdf]

foods

Table S1. Total phenolic contents, total flavonoids contents and antioxidant activities of free phenolics in legumes.

	Soybean	Vicia Faba	Kidney Bean
TPC(mg GAE/g DW)	2.04 ± 0.11[bc]	2.14 ± 0.08[b]	2.48 ± 0.04[a]
TFC(mg CAE/g DW)	0.71 ± 0.06 [bc]	0.78 ± 0.02[b]	1.41 ± 0.12[a]
ABTS(mg TE/g DW)	12.82 ± 0.49[b]	16.64 ± 0.34[a]	13.18 ± 0.36[b]
FRAP(mmol FE/g DW)	15.92 ± 1.41[b]	29.10 ± 1.81[a]	27.13 ± 1.77[a]

[a]Results are expressed as mean ± standard deviation of three replicates(n=3);

[b]Values followed by the different letters within the same line are significantly different ($P < 0.05$);

Table S2. Characterization of free phenolic constituents of legumes by UPLC-ESI-QTOF-MS2.

Peak no.	t_R (min)	λ_{max} (nm)	Formula	$[M-H]^-$ (m/z)	Major Fragment Ions (m/z)	Identification	Source
					**Phenolic acids and derivatives**		
1	3.66	285	$C_7H_6O_4$	153.0192	109.0268$[M-H-CO_2]^-$	Procatechuic acid[abc]	V
2	4.341	276,312	$C_{13}H_{16}O_9$	315.0707	153.0192$[M-H-C_6H_{10}O_5]^-$, 109.0282$[M-H-C_6H_{10}O_5-CO_2]^-$	Dihydroxybenzoic acid hexoside[a]	V
3	4.696	260,293	$C_7H_6O_4$	153.0211	109.0312$[M-H-CO_2]^-$	Dihydroxybenzoic acid[ab]	K
4	7.758	228,279	$C_7H_5O_3$	137.0219	92.9982$[M-H-CO_2]^-$	p-hydroxybenzoic acid[abc]	S, K, V
5	9.276	267,310	$C_{18}H_{16}O_8$	359.1118	197.0444$[M-C_9H_7O_3]^-$	Rosmarinic acid[a]	S
6	17.499	274,329		387.1646	163.1089,119.0316	Coumaric acid derivatives[a]	S, K
7	17.666	270	$C_9H_6O_3$	161.0818	133.8775$[M-CO]^-$, 117.0518$[M-CO_2]^-$	Hydroxycoumaric acid[ab]	V
8	18.747	219,270		387.1579	163.1111,119.0323	Coumaric acid derivatives[a]	K
9	19.102	272	$C_9H_8O_3$	163.0397	119.0504$[M-H-CO_2]^-$	p-coumaric acid[bc]	V
10	25.36	274	$C_{11}H_{12}O_5$	223.0583	164.0028$[M-H-CO_2-CH_3]^-$	Sinapic acid[abc]	K
					**Flavonoids**		
11	10.32	279	$C_{30}H_{26}O_{12}$	577.1334	289.0707$[M-H-C_{15}H_{12}O_6]^-$, 407.0694$[M-H-C_8H_{10}O_4]^-$, 425.0791$[M-H-C_8H_8O_3]^-$, 451.0903$[M-H-C_6H_6O_3]^-$, 125.0277$[M-H-C_{24}H_{20}O_8]^-$, 161.0245$[M-H-C_{21}H_{20}O_8]^-$	Procyanidin B[a]	K
12	11.85	279	$C_{15}H_{14}O_6$	289.0717	271.1518$[M-H-H_2O]^-$, 245.0807$[M-H-CO_2]^-$, 203.0663$[M-H-C_5H_{10}O]^-$, 179.0291$[M-H-C_6H_6O_2]^-$, 151.0389$[M-H-C_7H_6O_3]^-$, 109.0258$[M-H-C_9H_{12}O_4]^-$	Catechin[abc]	K
13	14.19	276	$C_{21}H_{32}O_{10}$	443.1953	101.0269,113.0244	penstemide[a]	S, V
14	15.731	246,279	$C_{21}H_{22}O_{10}$	433.116	343.0848$[M-H-C_3H_6O_3]^-$, 313.0695$[M-H-C_4H_8O_4]^-$,	Naringenin-8-c-β-D-glucoside[a]	S

					285.0760[M-H-$C_5H_8O_5$]$^-$, 271.0800[M-H-$C_6H_{10}O_5$]$^-$		
15	19.13	280	$C_{15}H_{10}O_6$	431.0905	311.1028[M-H-$C_6H_{12}O_2$]$^-$ 269.0829[M-H-C6H10O5]-	Vitexin[abc]	S, V
16	19.059	292	$C_{21}H_{22}O_{11}$	449.1084	287.0587[M-H-$C_6H_{10}O_5$]$^-$, 269.0460[M-H-$C_6H_{10}O_5$-H_2O]$^-$, 259.0642[M-H-$C_6H_{10}O_5$-CO]$^-$	Kaempferol hexoside[a]	S, K
17	19.233	278	$C_{21}H_{20}O_{10}$	431.1238	269.1[M-H-$C_6H_{10}O_5$]$^-$	Genistein[abc]	K
18	19.54	271	$C_{15}H_{14}O_6$	289.0717	109.1258[M-H-$C_9H_{12}O_4$]$^-$	Epicatechin[ac]	V
19	19.541	267	$C_{19}H_{30}O_8$	385.187	431.1895[M-H+HCOOH]$^-$, 223.1458[M-H-$C_6H_{10}O_5$]$^-$	Roseoside[a]	V
20	19.992	273	$C_{15}H_{10}O_5$	269.0656		Apigenin[ab]	V
21	23.07	279,325	$C_{10}H_{10}O_4$	193.0519	133.7612[M-H-$C_2H_4O_2$]$^-$	Ferulic acid[abc]	K
22	24.615	247,298	$C_{21}H_{20}O_9$	415.1051	461.1069[M+HCOOH-H]$^-$, 253.0520[M-$C_6H_{10}O_5$]$^-$	Daidzein[abc]	S
23	25.188	268,345	$C_{27}H_{30}O_{15}$	593.1584		Apigenin-6,8-bis-C-β-D-glucoside[a]	V
24	25.649	270	$C_{17}H_{24}O_{11}$	403.1778	223.0913, 179.1092	Loganin[a]	V
25	26.827	258,319	$C_{22}H_{22}O_{10}$	445.1114	491.1246[M-H+HCOOH]$^-$, 283.0626[M-H- $C_6H_{10}O_5$]$^-$	Glycitin[abc]	S
26	27.956	275,330	$C_{21}H_{22}O_{10}$	433.1123	271.0620[M-H-$C_6H_{10}O_5$]$^-$, 151.0061[M-H-$C_6H_{10}O_5$-C_6H_5O-C_2H_5]$^-$	Naringenin-7-O-glucoside[a]	S
27	28.897	260,327	$C_{21}H_{20}O_{10}$	431.0999	477.1030[M-H-HCOOH]$^-$, 269.0472[M-H-$C_6H_{10}O_5$]$^-$	Genistein[ac]	S
28	29.47	257,354	$C_{26}H_{28}O_{16}$	595.1341	301.0365[M-H-$C_6H_{10}O_5$-$C_5H_8O_4$]$^-$, 300.0296[M-H-$C_6H_{10}O_5$-$C_5H_9O_4$]$^-$	Quercetin-3-xyloside-7-glucoside[a]	K
29	30.64	260	$C_{15}H_{10}O_4$	253.0593	223.0371[M-H-CH_2O]$^-$,132.0212	Chrysin[ab]	S
30	31.393	265	$C_{21}H_{22}O_{10}$	433.1109	271.0611[M-H-$C_6H_{10}O_5$]$^-$, 151.0197[M-H-$C_6H_{10}O_5$-C_6H_5O-C_2H_5]$^-$	Naringenin Hexoside[a]	S
31	31.505	268	$C_{15}H_{12}O_5$	271.1573	151.0827[M-H-C_6H_5O-C_2H_5]$^-$	Naringenin[abc]	K
32	31.521	269	$C_{17}H_{24}O_{11}$	403.1598	223.0953,179.0595	Loganin[a]	V
33	31.78	261,328	$C_{16}H_{12}O_5$	283.0737	268.0400[M-H-CH_3]$^-$,240.0515	Glycitein[abc]	S
34	32.17	245,294,333		637.1428	577.1161,457.0958,431.0781,413.0722, 353.0463	Vitexin rhamnoside derivatives[a]	S

35	32.53	263,353	$C_{27}H_{30}O_{16}$	609.142	301.0337[M-H-$C_6H_{10}O_5$-$C_6H_{10}O_4$]$^-$	Rutin[ac]	K
36	32.8	265,348	$C_{21}H_{20}O_{12}$	463.0902	301.0400[M-H-$C_6H_{10}O_5$]$^-$, 300.0298[M-H-$C_6H_{11}O_5$]$^-$, 271.0299[M-H-$C_7H_{12}O_6$]$^-$	Hyperoside[ac]	K
37	33.271	260,329	$C_{15}H_{10}O_5$	269.0487		Genistein[abc]	S
38	33.85	263,346	$C_{15}H_{10}O_4$	253.0543	135.0182[M-H-C_8H_6O]$^-$, 107.1126[M-H-$C_9H_6O_2$]$^-$	Daidzein[abc]	S
39	34.72	235,271	$C_{16}H_{12}O_5$	283.0596	268.1[M-H-H_2O]$^-$ 240.0453[M-H-CH_3-CO]$^-$	Glycitein[abc]	S
40	35.271	266,346	$C_{21}H_{20}O_{11}$	447.1032	285.0488[M-H-$C_6H_{10}O_5$]$^-$	Kaempferol hexoside[ab]	K
41	35.5	246,285	$C_{15}H_{12}O_5$	271.1028	150.9 [M-H-C_6H_5O-C_2H_5]-	Naringeninabc	S
42	35.72	266	$C_{15}H_{10}O_5$	269.0469		Genistein[ab]	S, K
43	35.836	260,327	$C_{15}H_{10}O_5$	269.0474	225.0537[M-H-CO_2]$^-$	Apigenin[ab]	S
44	36.27	267	$C_{24}H_{18}O_{14}$	533.1604	489.1030[M-H-CO_2]$^-$, 285.0437[M-H-C_3O_2-$C_6H_8O_6$]$^-$	Kaempferol 3-O-(6''-O-malonyl) glucuronic acid[a]	K
45	37.34	230,267	$C_{15}H_{10}O_7$	301.0353	151.0138[M-H-$C_8H_6O_3$]$^-$	Quercetin[abc]	K
46	37.481	260,331	$C_{23}H_{23}O_{11}$	473.1111	519.0327[M-H+HCOOH]$^-$, 269.0475[M-C_2H_3O-$C_6H_{10}O_5$]$^-$	Acetyl genistein[a]	S
47	37.707	262	$C_{20}H_{22}O_{10}$	421.2097	289.1649[M-H-$C_5H_8O_4$]$^-$, 467.2139[M-H+HCOOH]$^-$	Catechin-7-furan arabinoside[ab]	S
48	40.05	265,365	$C_{15}H_{10}O_6$	285.0414	108.0191,159.0413,239.0322	Kaempferol[ab]	K

S: soybean; V: vicia faba; K: kidney bean.

[a]Compared with literatures.

[b]Compared with MSn data, data bases and/or characteristic UV spectra.

[c]Compared with an authentic standard.

Table S3. Correlation analysis of phenlic and flavonoid contents and antioxidant activity.

	TPC	TFC	FRAP	ABTS
TPC	1	0.845**	0.455	0.414
TFC		1	0.977**	0.929**
FRAP			1	0.733**
ABTS				1

Pearson's correlation coefficient (R2); * $p < 0.05$, ** $p < 0.01$.

Table S4. pH in different time of colonic fermentation.

	0h	1h	3h	6h	12h	24h	36h	48h
Soybean	6.88	6.81	6.37	6.6	6.6	6.67	6.44	6.24
Vicia faba	6.82	6.59	5.72	5.13	4.53	4.36	4.19	4.13
Kidney bean	6.92	6.77	5.92	4.94	4.22	4.2	4.03	4.01

Table S5. The optimized MS parameters of standard substance in the quantitive analysis.

Compounds	t_R (min)	Calibration Curve Equation	R^2	Precursor Ion (m/z)	Product Ion (m/z)	Fragmentor Voltage (V)	Collision Energy (eV)	LOD (ng/mL)	LOQ (ng/mL)
**Phenolic acids**									
p-hydroxybenz-oic acid	6.62	y = 4133.3x + 843.45	0.9992	137	92.9	85	8	24.85	82.85
procatechuic acid	4.303	y = 5265.9x - 1071.4	0.994	153	108.9	80	10	9.96	33.19
ferulic acid	12.974	y = 394.41x + 23.33	0.9947	193.1	133.8	100	6	13.2	44
chlorogenic acid	8.435	y = 7581.8x - 1180.6	0.999	353.1	190.8	100	15	2.87	9.56
sinapic acid	13.585	y = 1555.2x + 63.123	0.9995	223.1	164	105	12	6.21	20.69
gallic aicd	1.891	y = 2291.3x + 229.97	0.9992	169	125.1	100	6	47.32	137.72
p-coumaric acid	11.525	y = 7941.3x - 1322.9	0.9936	163.1	119	95	10	12.08	40.26
Isoflavones									
daidzein	19.056	y = 2156.6x + 320.88	0.9986	253.1	132	180	36	1.05	3.5
daidzin	12.668	y = 3373.3x - 336.87	0.9987	415.1	252.2	190	25	0.72	2.4
genistein	21.033	y = 3629.3x + 616.13	0.9966	269	133.1	150	32	0.98	3.28
genistin	14.636	y = 4743.8x + 601.53	0.9984	431.1	269.1	160	20	1.15	3.84
glycitein	19.85	y = 4410.7x + 47.148	0.9996	283.1	268.1	130	12	2.78	9.27
glycitin	13.484	y = 3090.2x + 186.5	0.9989	445.1	282.2	190	23	0.53	1.75
Flavones									
biochanin A	23.851	y = 20743x + 7675	0.9984	283.1	268.1	130	20	0.13	0.44
quercetin	20.05	y = 6893.7x - 423.82	0.999	301	151	135	19	1.74	5.79
hyperoside	15.924	y = 12368x + 1444.8	0.9989	463.1	300.1	170	23	0.29	0.99
rutin	16.253	y = 4679.8x - 175.59	0.9988	609.1	300	225	40	0.86	2.87
vitexin	14.815	y = 25526x + 46.389	0.9991	431.1	311.1	160	18	0.54	1.82
Flavanones									
naringenin	20.164	y = 8523.2x - 44.704	0.999	271.1	150.9	120	20	0.73	2.46
Flavanes									
catechin	7.521	y = 390.99x + 8.3217	0.9992	289.1	109.1	135	19	22.49	74.96
